# Efficacy and safety of ucha-shinki-hwan on korean patients with cold hypersensitivity in the hands and feet: Study protocol clinical trial (SPIRIT Compliant)

**DOI:** 10.1097/MD.0000000000019110

**Published:** 2020-02-21

**Authors:** Youme Ko, Seung-Ho Sun, Ho-Yeon Go, Jin-Moo Lee, Jun-Bok Jang, Hyun-Kyung Sung, Bo-Hyoung Jang, Chan-Yong Jeon, Seong-Gyu Ko

**Affiliations:** a Department of Science in Korean Medicine, Graduate School, Kyung Hee University, Kyungheedae-ro, Dongdaemun-gu; b Institute of Safety and Effectiveness Evaluation for Korean Medicine, Kyung Hee University, Seoul; c Department of Korean Internal Medicine, College of Korean Medicine, Sangji University, Sangjidae-gil, Wonju-si, Gangwon-do; d Department of Korean Internal Medicine, College of Korean Medicine, Semyung University, Semyeong-ro, Jecheon-si, Chungcheongbuk-do; e Department of Korean Gynecology, College of Korean Medicine, Kyung Hee University, Kyungheedae-ro, Dongdaemun-gu, Seoul; f Department of Pediatrics, College of Korean Medicine, Hospital of Semyung University, St. Sangbang, Chungju City, Chungcheongbuk-do; g Department of Korean Internal Medicine, College of Korean Medicine, Gachon University, Seongnamdae-ro, Sujeong-gu, Gyeonggi-do, Republic of Korea.

**Keywords:** cold hypersensitivity, cold intolerance, herbal medicine, Korean medicine, ucha-shinki-hwan

## Abstract

**Background:**

Cold hypersensitivity in the hands and feet (CHHF) is a common complaint in Asian female population especially in Korea. Due to the symptoms of CHHF the quality of individual's daily life can be degraded. Ucha-Shinki-Hwan (UCHA) is widely used in the treatment of various diseases including CHHF by harmonizing Yin and Yang, and improving the vitality of whole body. However, the efficacy of UCHA as a treatment option of CHHF has not been assessed in trials. Thus, we aimed to investigate the efficacy and safety of UCHA in Korean women with CHHF through this trial.

**Methods:**

This study will be an exploratory, randomized, double-blind, placebo-controlled, parallel-group, multicenter trial. Korean women aged 19 to 59 years who complaint with CHHF will be enrolled from 5 university affiliated Korean medicine hospitals. A total of 164 subjects will be randomly assigned to a treatment group (UCHA) or a placebo group at a 1:1 ratio. The subjects will receive 2.5 g of either UCHA or placebo three times a day for 8 weeks. The primary outcome will be evaluated with the visual analog scale score of CHHF. The secondary outcome measures will be changes in skin temperature in extremities as measured by using a thermometer and the Korean version of the World Health Organization Quality of Life Scale Abbreviated Version.

**Discussion:**

This study will be the first trial to explore the efficacy and safety of UCHA for CHHF patient. This will provide meaningful clinical information on herbal medicine treatment of CHHF and a clinical evidence for planning a full randomized clinical trial.

**Disclosures and acknowledgments:**

The authors report no competing interests.

**Trial registration:**

This study is registered at ClinicalTrials.gov, ID: NCT03790033. Registered on (31 December 2018)

**Protocol version:**

The final approved version of the trial protocol is V1.3. (25 January 2019)

## Abstract 2

Supplemental Digital Content is available in the text

## 1 Introduction

Cold hypersensitivity in the hands and feet (CHHF) is a condition of thermal hyperalgia in which patient feels a sense of coldness in the extremities to a greater extent than unaffected people in any environment, with worsening discomfort at lower temperatures.^[1]^ CHHF patients mostly complain of discomfort when working with their hands, and a low quality of life due to the inconvenience of wearing socks or gloves in hot weather.^[2]^ It is mostly diagnosed by patient's subjective symptoms and experiences, which has no clear conception in Western medicine.^[3]^ According to a previous observational study, cold hypersensitivity is frequent in Asians, particularly among females compared to males with an approximate ratio of 3:2.^[4]^

In Korean medicine, CHHF is considered a treatable symptom that is caused by 3 types of pathogens. First, there is an invasion of pathogens that cause a cold through external pathogenic factors, affecting the whole body. The other is the disharmony of specific internal organs, such as deficiency of spleen yang, kidney yang, blood, or Qi. The last is the concurrent occurrence of cold and heat syndrome due to the lack of stomach harmony and adverse Qi.

Ucha-Shinki-Hwan (UCHA) is a frequently prescribed herbal formula by East Asian medicine practitioners, mostly in Korea, Japan, and China.^[5]^ It is mentioned in a medical book called Ji- sheng Fang (A Book of Formulas to Promote Well-Being), written in the 13th century by Master Yan.^[6]^ It is used to treat kidney deficiency syndrome and lower limb dysfunction by balancing Yinyang and removing dampness and turbidity. Previous research has reported that it has beneficial effects in various diseases including chemotherapy-induced neuropathy with coldness,^[7–9]^ diabetic neuropathy,^[10]^ and overactive bladder.^[11–12]^ Our research team also performed an in vitro study on the inhibitory effect of UCHA on cold-induced response in human dermal microvascular endothelial cells (HDMEC) and observed the inhibitory effects on the contraction of cold-exposed HDMEC by targeting RhoA activation.^[13]^

Although UCHA is typically prescribed in the treatment of various symptoms and concomitant cold extremity, a randomized clinical trial (RCT) has not been performed yet to assess the clinical efficacy of UCHA in the treatment of CHHF. Therefore, this exploratory, randomized, placebo-controlled, double-blind trial aims to evaluate the efficacy and safety of UCHA and to examine the feasibility of a full RCT for the treatment of CHHF in Korean female patients.

## 2 Methods

### 2.1 Objectives

The aim of this trial is to evaluate the efficacy and safety of UCHA in Korean female patients with CHHF, using an exploratory RCT protocol that includes examination of the severity of CHHF, skin temperature (ST), quality of life, and safety.

### 2.2 Study design and setting

This pilot, randomized, double-blind, placebo-controlled, parallel-group, multi-center clinical trial will be conducted in 5 university-affiliated Korean medicine hospitals, namely Gachon University Gil Korean Medical Hospital, Korean Medical Hospital of Sangji University, Semyung University Second Affiliated Korean Medical Hospital at Chungju, Kyung Hee University Korean Medical Center, and Kyung Hee University Korean Medicine Hospital at *Gangdong*.

Written informed consent will be obtained from the subjects, after giving detailed explanation and they will be screened up to 7 days prior to randomization. Once assigned to the subject identifier, they will be administered investigational products (IPs) for 8 weeks and asked to visit the enrolled trial site every 4 weeks for efficacy and safety examination. At 4 weeks after post treatment visit, follow-up visit will be scheduled. The participants will be asked to return any remaining IPs at each visit to monitor adherence, in order to calculate medication compliance. During the trial, the participants will be prohibited from administering any medications which can impact CHHF. The methodology of the trial is based on the consolidated standards of reporting trials guidelines and standard protocol items: recommendations for interventional trials checklist (see additional file 1). Table 3 presents a schematic flow diagram of the study.

### 2.3 Participants

#### 2.3.1 Inclusion criteria

The inclusion criteria of this study are as follows: patients aged 19 to 59 years who present with CHHF; CHHF score higher than 40 points on the 100-mm visual analog scale (VAS); thermal differences between the palm (PC8) and the upper arm (LU4) may be higher than 0.3°C and differences between anterior of thigh (ST32) and the top of the foot (LR3) may be higher than 2.0°C; symptoms of CHHF in normal temperatures when most individuals do not feel cold or having symptoms of extremely cold hands on cold temperature exposure or individuals who are on the return to a warmer environment but, their cold hands have not completely returned to a normal temperature. Voluntary participation with a signed informed consent form after a detailed explanation of the trial has been obtained.

#### 2.3.2 Exclusion criteria

The exclusion criteria of the UCHA trial are provided in Table 1. Volunteers who meet any of the conditions cannot enroll to this study.

**Table 1:**
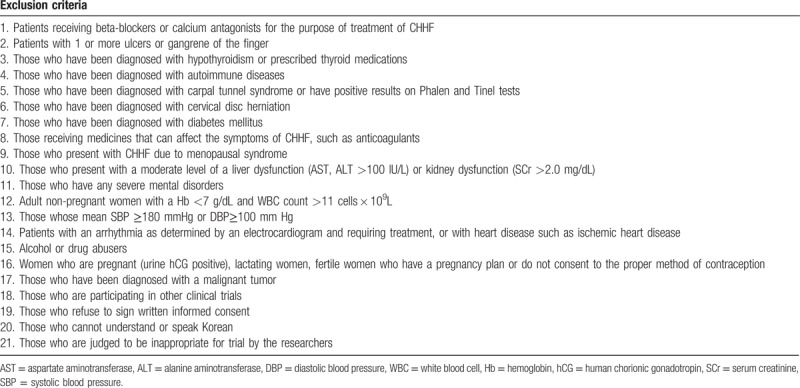
Exclusion criteria of the trial.

#### 2.3.3 Participant withdrawal criteria

The trial would be discontinued if participants met any of the criteria summarized in Table 2. The subjects who are withdrawn after randomization will be followed-up for outcomes. Reasons for withdrawal will be documented in case report forms (CRFs) and data will be analyzed using the intention-to-treat (ITT) principle. Each site clinical research coordinator will be responsible for making next visit reminder phone calls to prevent participant dropout.

**Table 2:**
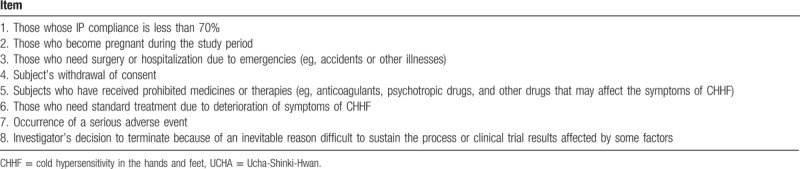
Withdrawal criteria of the UCHA study.

**Table 3:**
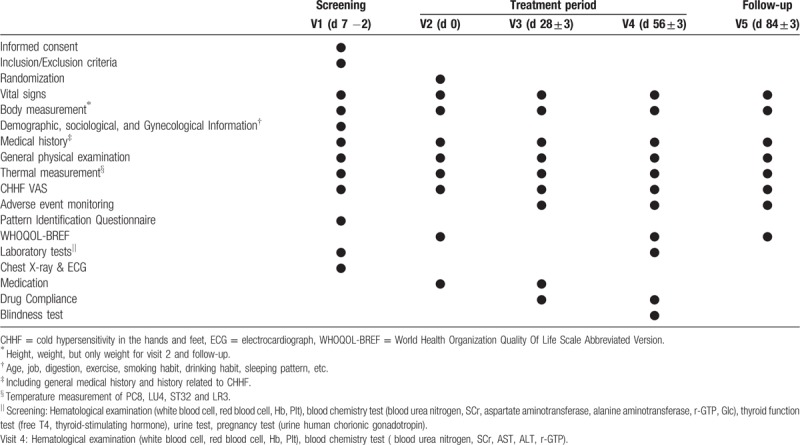
Study flow of the trial.

### 2.4 Randomization

Suitable participants, who agree to participate in this RCT, will be randomly assigned to a UCHA group or a placebo group at a 1:1 ratio. A total of 164 eligible participants each will be allocated to the UCHA and placebo groups. An internet-based research randomizer (IRR) developed by the contract research organization (CRO), the Institute of Safety and Effectiveness Evaluation for Korean Medicine (ISEE), will perform the assignment. A random order will be generated by the CRO's independent professional statistician using the R software (The R Foundation for Statistical Computing, Vienna, Austria) and stratified by the hospital using random block sizes of 2 and 4 based on the allocation code provided by the R software.

### 2.5 Blinding

Both the researchers and subjects will be blinded to the assigned IP. SHINHWA Pharmaceuticals will package, label the randomization code number on the IP, and distribute to trial sites. Each subject will receive the IRR-assigned IP by independent research pharmacists or assistants who are also blinded to the randomization, in each hospital.

### 2.6 Procedure

#### 2.6.1 Recruitment

Participants will be recruited from 5 university-affiliated Korean medicine hospitals located in various administrative areas in Korea. Each site will recruit the participants who have voluntarily visited each trial site for enrollment or through public advertising.

#### 2.6.2 Study schedule

The items to be measured at each visit are provided in Table 3.

### 2.7 Interventions

After randomization, the participants will be prescribed 2.5 g of UCHA or placebo 3 times a day after 30 minutes of each meal for 8 weeks.

UCHA is manufactured by Tsumura Co. (Tokyo, Japan). UCHA granulated extract contains 1.7 g of Rehmannia glutinosa Radix, 1.0 g of Achyranthes bidentata Blume Radix, 1.0 g of Cornus officinalis Fructus, 1.0 g of Dioscorea batatas Decne. Rhizoma, 1.0 g of Plantago asiatica Semen, 1.0 g of Alisma orientale Rhizoma, 1.0 g of Poria cocos Sclerotium, 1.0 g of Paeonia suffruticosa Cortex, 0.3 g of Cinnamomum cassia Cortex, and 0.3 g of Aconitum carmichaelii Tuber. It will be extracted from the raw materials described above and concentrated to 2.5 g per dose.

Placebo is manufactured by SHINHWA Pharmaceuticals (Daegu, Korea). The granulated extract contains 2.25 g of lactose, 0.175 g of dextrin, and 0.075 g of caramel coloring per dose. It has a similar color, shape, weight, flavor, and taste to that of UCHA.

Both IPs are packaged and distributed to trial sites by SHINHWA Pharmaceuticals.

### 2.8 Outcome

#### 2.8.1 Primary outcome measure

##### 2.8.1.1 VAS

The VAS score (ranging from 0 mm as no discomfort to 100 mm as the maximum coldness imaginable) will be used to assess the severity of CHHF at every visit.^[14]^

#### 2.8.2 Secondary outcome measures

##### 2.8.2.1 ST: Thermometer measurement

After 20 minutes of relaxation at 24 ± 2°C, the ST will be measured by a thermometer (Testo 835-T1, Lenzkirch, Germany) at acupoints PC8, LU4, ST32, and LR3 at visit 2, 3, 4, and follow-up.

##### 2.8.2.2 World Health Organization Quality Of Life Scale Abbreviated Version (WHOQOL-BREF)

The Korean version of the WHOQOL-BREF consists of a 26-item questionnaire that measures 5 domains of an individual's quality of life, namely general quality of life; psychological, physical, and environmental health; and social relationships.^[15]^ It will be conducted at visits 2, 4, and follow-up.

#### 2.8.3 Safety outcome measure

The safety assessment will be performed for all subjects who have been administered IP at least once. The subjects’ vital signs and general physical condition will be examined at every visit. Hematological examination (white blood cell, red blood cell, hemoglobin, and platelet), blood chemistry test (blood urea nitrogen, creatinine, aspartate aminotransferase, alanine aminotransferase, gamma-glutamyl transpeptidase) will be performed at screening and visit 4. Blood glucose test, thyroid test (free T4 and thyroid-stimulating hormone), urine analysis, and pregnancy test (urine human chorionic gonadotropin) will be conducted at screening visit. The occurrence of adverse events (AEs) will be checked at visit 3, 4, and 5.

#### 2.8.4 Compliance calculation

At visit 2 and 3, a bag of IP, which includes 93 aluminum foil packages, will be provided to the subjects. They will be asked to return the remaining investigational medications. The rate of compliance will be calculated as compliance (%) = ([93 - remaining products]/expected intake) × 100. Clinical trials will be continued only if compliance is equal to or greater than 70%.^[16]^

#### 2.8.5 AE reporting

The investigators should educate the subjects to inform about any AEs that occur after IP administration. All AEs that occur after the start of this trial should be recorded in the CRF whether or not they are related to the IP and should also be evaluated for causal relationships. In the event of any irregular medical conditions, such as serious AEs, the principal investigator of the site will contact the CRO and sponsors immediately to process the codebreaking in accordance with standard operating procedures (SOPs). They are also required to notify the Institutional Review Board and regulatory authorities within 24 hours.

#### 2.8.6 Sample size calculation

The change in the CHHF VAS score between baseline and post treatment will be used as the primary outcome measure. The hypothesis is follows: 
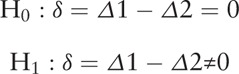


Δ1: Mean change in the CHHF VAS score between baseline and post treatment in the UCHA group

Δ2: Mean change in the CHHF VAS score between baseline and post treatment in the placebo group

Based on the prior research,^[14]^ the expected mean CHHF VAS score difference in the SDT group is 10.93, and the pooled standard deviation (SD) is estimated to be 22.28. Considering a dropout rate of 20%, we determine to recruit 82 subjects per arm to calculate the treatment efficacy and to offer the power calculation for a future large-scale RCT. Each site will recruit the participants competitively.

#### 2.8.7 Statistical analyses

##### 2.8.7.1 Efficacy assessment

For the efficacy assessment of this trial, both ITT and per-protocol (PP) analyses will be performed. Missing data will be adjusted using the last-observation-carried-forward (LOCF) imputation method on ITT analysis. The continuous variables will be expressed as the mean ± SD and the nominal variables will be reported as percentages. The ITT method will include all randomized subjects, regardless of any protocol violations or trial dropouts. The PP method will include only the subjects who have completed the whole study period without any major protocol violations and who have medication compliance rate equal to or greater than 70%. Missing values from the efficacy analysis will be modulated by applying the LOCF. The baseline demographic information will be compared by either a chi-square test for nominal variables or an analysis of variance (ANOVA) for continuous variables. For the within-group analysis, the primary and secondary outcome variables will be assessed using a paired *t* test. The between-groups analysis after 8 weeks of administration will be performed using a repeated-measure ANOVA for the primary outcome variables, and a paired *t* test for the mean difference in the VAS score between the SDT and placebo groups. As a secondary outcome variable, the mean differences in ST and WHOQOL-BREF scores between the groups will be analyzed using a repeated-measure ANOVA for ST and an ANOVA and post-hoc analysis for the WHOQOL-BREF scores. For all non-normal distribution data, a nonparametric statistical test will be performed. SPSS for Windows version 25.0 (SPSS Inc., Chicago, IL) will be used for statistical analysis. The statistical significance level will be set at *P* < .05.

##### 2.8.7.2 Safety assessment

Safety analysis will be conducted for all subjects who will be randomized and would visit at least once after screening. Safety-related measures will be analyzed using the ITT method. The safety data will be stratified according to the symptoms.

### 2.9 Data management and monitoring

All records will be collected in paper CRF documents. To protect personal confidentiality, the files would be stored in a secure and locked place without any personally identifiable information. After trial completion, 2 independent technicians will perform data entry for promoting the data quality. After finishing the data entry and dealing with the queries by range checking, the database will be locked and analyzed by independent professional statistician of ISEE. Site PIs will have direct access to the final data sets from their own sites. Monitoring will be performed by ISEE and will begin after the first participant completes the entire course of the study. All institutions conducting trials will be monitored in accordance with the SOPs during the trial. No auditing will be performed for this trial.

### 2.10 Ethics approval and consent to participate

This trial has been approved by the IRBs of five hospitals (Gachon University Gil Korean Medical Hospital: 18–102, Kyung Hee University Oriental Medical Center: KOMCIRB-2018–09–006, Kyung Hee University Korean Medicine Hospital at Gangdong: KHNMCOH2018-09-001, Korean Medical Hospital of Sangji University: SJIRB-18-003 and Semyung University Second Affiliated Korean Medical Hospital at Chungju: SMCJH 1810-06). The trial will be conducted in accordance with the Helsinki declaration and the Good Clinical Practice Guidelines of the Korea Food and Drug Administration. All participants will be signed the written informed consent document and received the copied consent documents prior to any trial procedures.

If any protocol changes which may have impacted the trial, it must approve by the site IRB prior to implementation. After approval, each site PI should be notified and trained the site staffs before initiating the trial.

The results of this UCHA trial will be disseminated through scientific journals or poster presentations at medical conferences. The public access to any trial data set is still unplanned.

## 3 Discussion

This pilot study is aimed to explore the clinical efficacy and safety of UCHA to treat CHHF patients. Recently, CHHF has been observed to affect the quality of daily life of individuals in East Asian countries, which can be considered as the women's burden of disease. In Korean medicine, CHHF has been known as a cold-related symptom and pattern, which requires careful attention to prevent symptom worsening and further pattern progression into a severe concomitant illness. Traditional herbal medicine is commonly used in the treatment of CHHF in Korea. However, the evidence of efficacy and safety is not investigated rigorously. Therefore, the establishment of systemic treatment option as well as its guideline for CHHF is essential.

This trial is the first well-designed RCT to evaluate the efficacy and safety of UCHA compared to placebo in patients with CHHF in Korea, which is the major strength of this study. However, this trial has several limitations. First, due to the diversity in IP manufacturing companies, IPs cannot be manufactured with a remarkably same appearance with each other. To minimize the unblinding, we make the samples several times to develop a placebo that cannot be noticed by patients by arranging researcher meetings. The second is the limited use of scientific quantitative outcome measurements. In Western medicine, the assessment of cold intolerance-related diseases typically involves infrared thermography, cold stress test (CST), and heart rate variability analysis to quantify the severity of the disease. However, previously, our team performed a study^[17]^ on CHHF with herbal medicine, Danggui-SayukGa-Osuyu-Saenggang-tang, and conducted the CST, which is a more accurate measuring method to establish the efficacy of herbal medicine on CHHF. However, the study did not reveal any significant difference on groups after treatment and it had a higher risk of assessment bias during the performing test; we, therefore, had to exclude it from the outcome measurement.

Despite these limitations, as the first trial to examine the efficacy and safety of UCHA on CHHF patients, we expect that the study findings may provide informative evidence to design a confirmative, large-scale RCT in the near future. Moreover, it will help practitioners to utilize UCHA as a therapeutic regimen for CHHF.

### 3.1 Trial status

Participant recruitment has begun on January 31, 2019, and 72 participants have been recruited until now. And it will complete in May 2020.

## Author contributions

**Conceptualization:** Seung-Ho Sun, Ho-Yeon Go, Jin-Moo Lee, Jun-Bok Jang, Hyun-Kyung Sung, Bo-Hyoung Jang, Chan-Yong Jeon

**Methodology:** Seung-Ho Sun, Ho-Yeon Go, Jin-Moo Lee, Jun-Bok Jang, Bo-Hyoung Jang, Chan-Yong Jeon

**Project administration:** Chan-Yong Jeon, Seong-Gyu Ko

**Statistical Consultation:** Bo-Hyoung Jang

**Supervision:** Chan-Yong Jeon, Seong-Gyu Ko

**Writing – original draft:** Youme Ko

**Writing – review & editing:** Ho-Yeon Go, Jin-Moo Lee, Jun-Bok Jang, Hyun-Kyung Sung, Chan-Yong Jeon, Seong-Gyu Ko

## Supplementary Material

Supplemental Digital Content
