# Supplementary material for: Efficacy and safety of ucha-shinki-hwan on korean patients with cold hypersensitivity in the hands and feet: Study protocol clinical trial (SPIRIT Compliant)
Source: Medicine (Baltimore). 2020 Feb 21;99(8):e19110. doi: 10.1097/MD.0000000000019110 (PMC7034626; doi:10.1097/MD.0000000000019110)
Supplement: Supplemental Digital Content [file medi-99-e19110-s001.docx]

**[Apendix] Informed Consent Form (ver 1.1)**

**Study Title: Efficacy and Safety of Ucha-Shinki-Hwan on Korean Patients With Cold Hypersensitivity in the Hands and Feet-Double Blinded, Randomized, Multicenter, Placebo Controlled Clinical Trial**

**I have read the foregoing information, or it has been read to me. □**

**I have had the opportunity to ask questions about it and any questions that I have asked have been answered to my satisfaction. □**

**I was also informed that I can withdraw the agreement and receive appropriate treatment if any adverse event occurs. □
I agree that I will cooperate with study investigators and talk to study investigators about any side effects that I have while taking part in the study. □
I consent voluntarily to participate as a participant in this research. □
A copy of this ICF has been provided to the participant. □**

**Participant**

**Print Name __________________ Signature ___________________ Date ____________________**

**Legal representative (if necessary)**

**Print Name __________________ Signature ___________________ Date ____________________**

(Relationship)

**Witness (if necessary)**

**Print Name __________________ Signature ___________________ Date ____________________**

**Researcher/person taking the consent**

**Print Name __________________ Signature ___________________ Date ____________________**
